# Supplementary material for: Which osteoarthritic gait features recover following total knee replacement surgery?
Source: PLoS One. 2019 Jan 25;14(1):e0203417. doi: 10.1371/journal.pone.0203417 (PMC6347391; doi:10.1371/journal.pone.0203417)
Supplement: S1 Fig — (PDF) [file pone.0203417.s001.pdf]

S1. Fig - Principal component reconstruction of the 18 biomechanical gait features retained for analysis

Below are principle component reconstructions are displayed which can be used to interpret the biomechanical feature represented by each component. Columns show the biomechanical features represented by each principal component (PC). Two common visualisations used for interpretation – representative extremes (top row) and single component reconstruction (bottom row) are plotted. The top row contains the raw waveforms (light grey, solid lines) for the 30 non-pathological participants and 30 participants with late stage knee osteoarthritis. The waveform of the subject with the 95th percentile (thick, dashed red line) principal component (PC) score, the 5th percentile (thick, solid blue line) PC score are plotted. A consistent sign convention was used such that low PC values correspond to features seen predominantly in osteoarthritic subjects. In the middle row the squared factor loadings are plotted, which correspond to the proportion represented by the PC at each point of the gait cycle, where a value of 1 corresponds to 100% of variance. In the bottom row, the raw waveform of the same 95th percentile (dashed red) and 5th percentile (solid blue) subjects are reconstructed by multiplying the PC of the subject by the eigenvector.

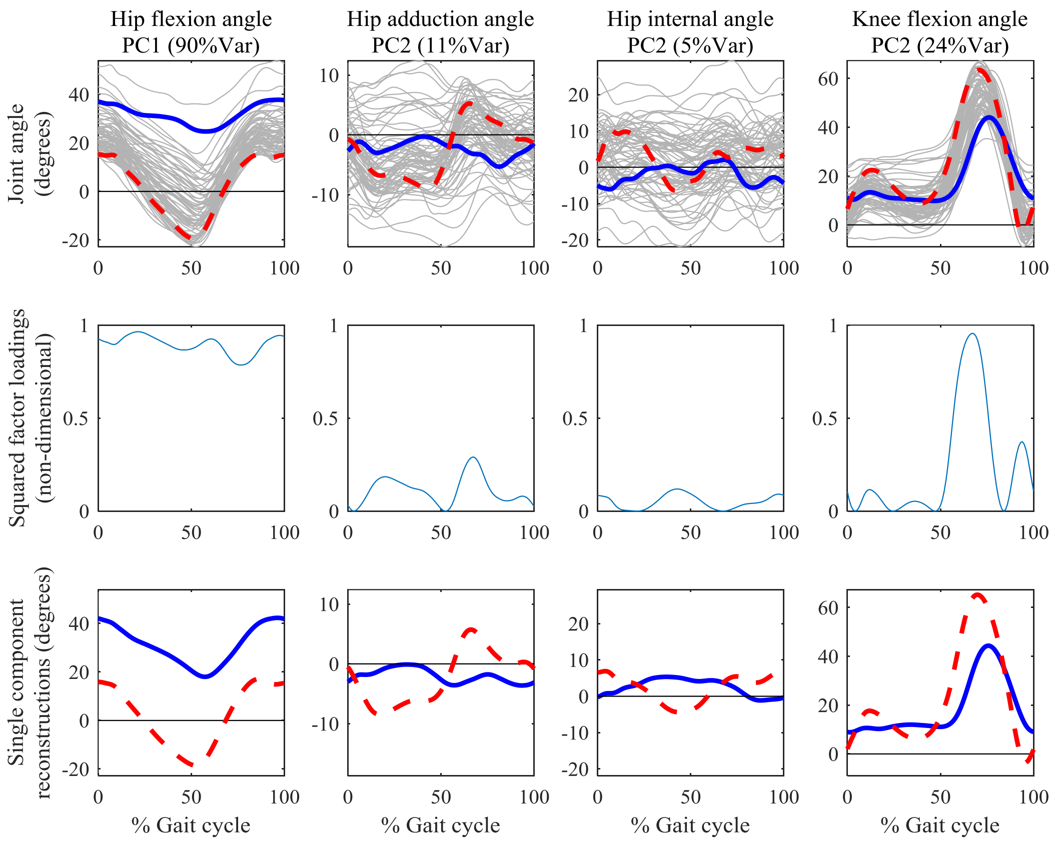

Fig. A) Principal component reconstruction of joint kinematic features during gait

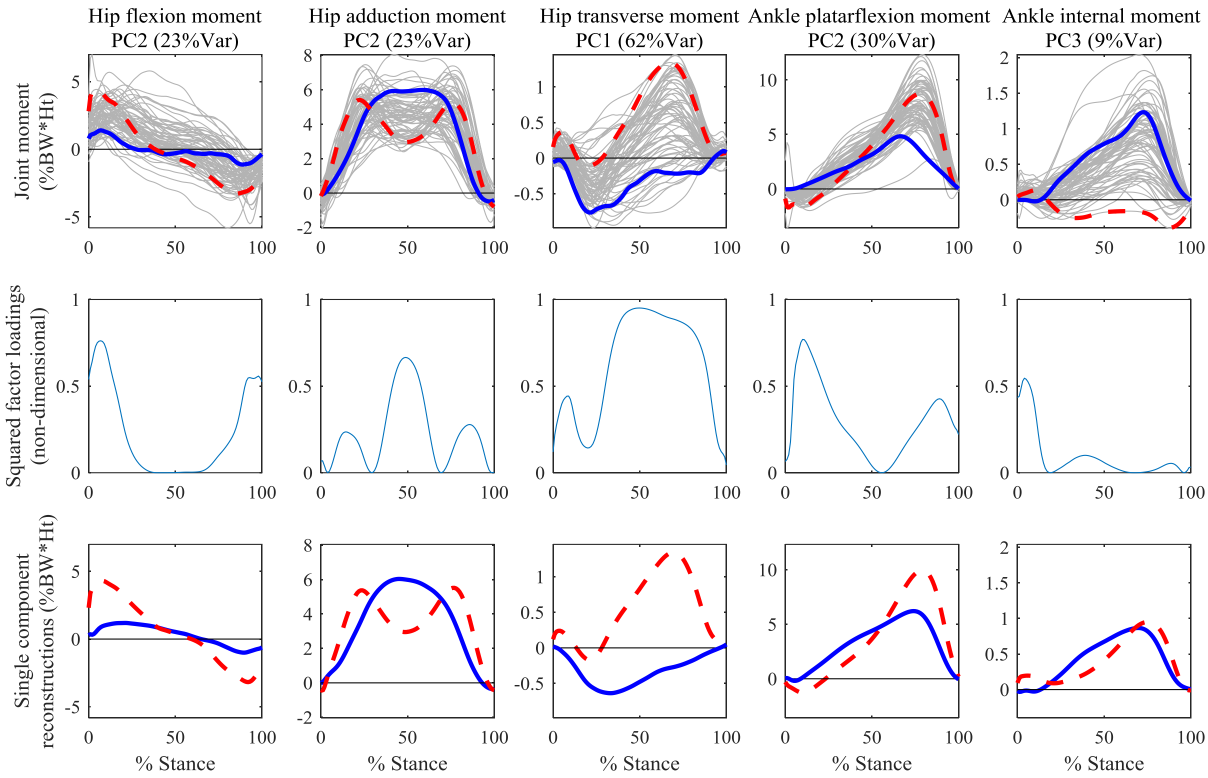

Fig. B) Principal component reconstruction of hip and ankle joint moment features during gait.

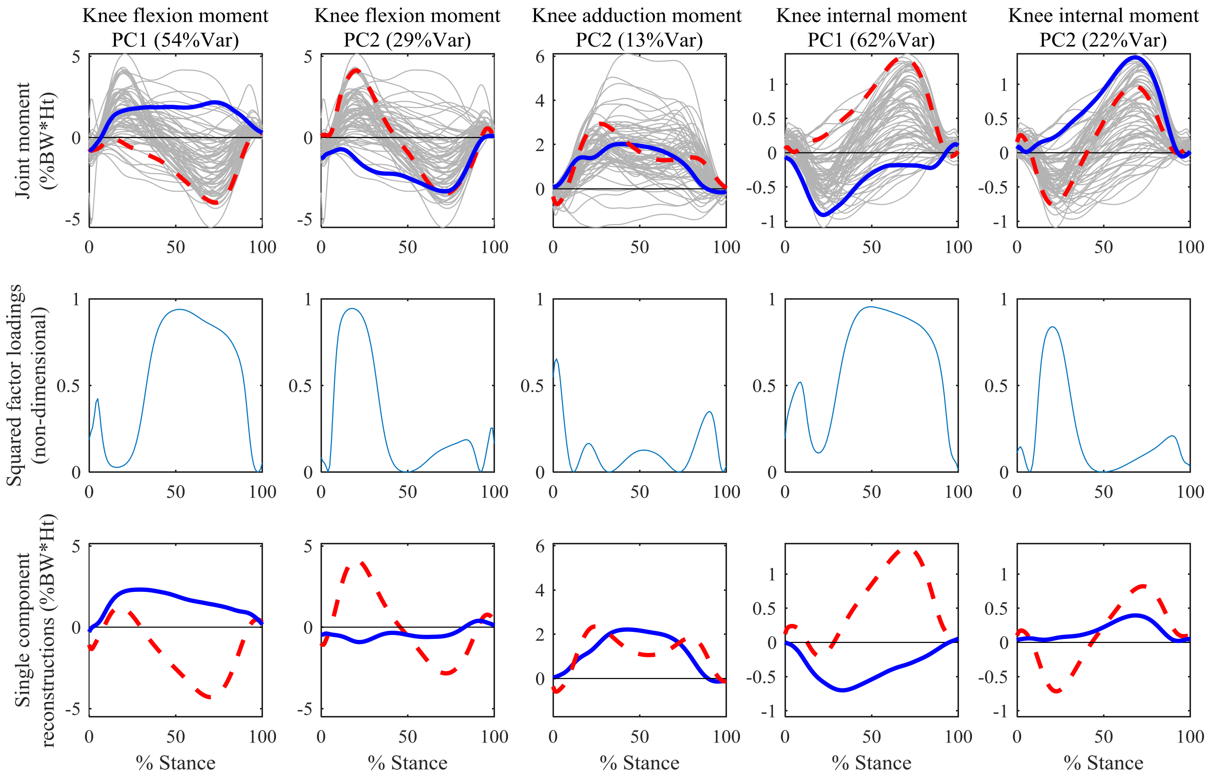

Fig. C) Principal component reconstruction of knee joint moment features during gait.

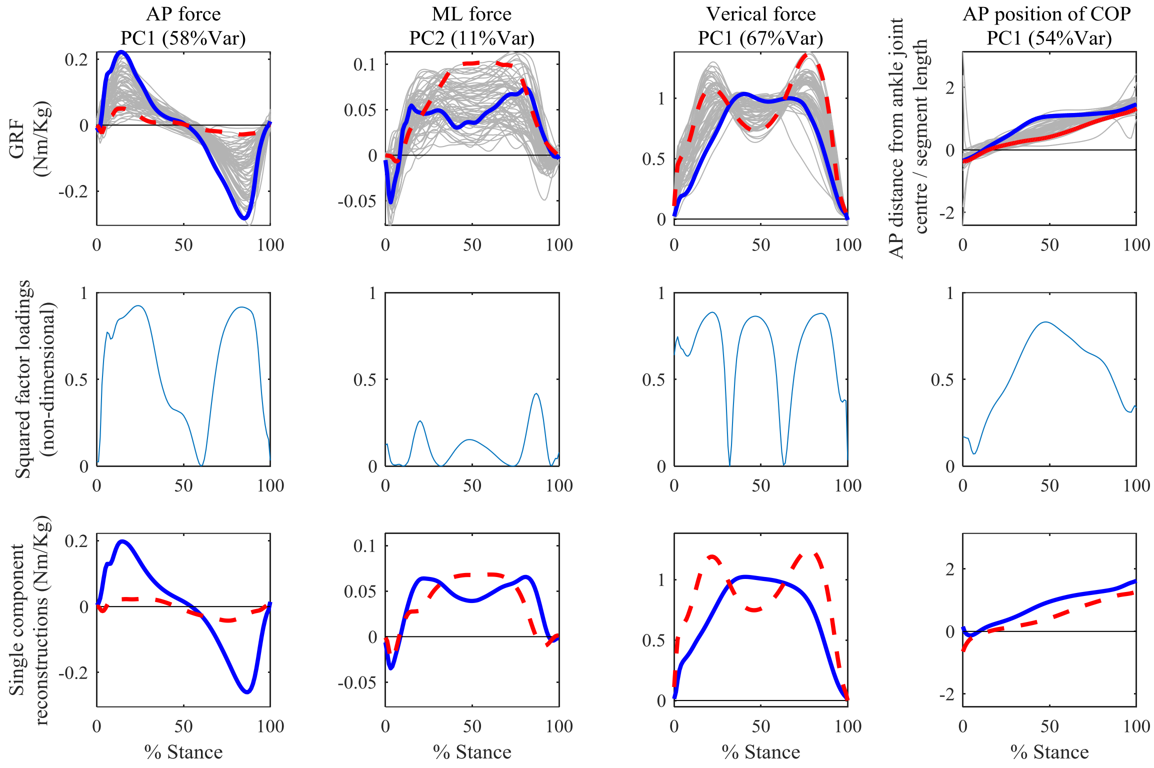

Fig. D) Principal component reconstruction of ground reaction force and centre of pressure features during gait.
